# Evaluation of the geometric and dosimetric accuracies of deformable image registration of targets and critical organs in prostate CBCT‐guided adaptive radiotherapy

**DOI:** 10.1002/acm2.14490

**Published:** 2024-09-13

**Authors:** Hussam Hameed Jassim, Hassan Ali Nedaie, Nooshin Banaee, Ghazale Geraily, Ali Kazemian, Danial Seifi Makrani

**Affiliations:** ^1^ Department of Medical Physics and Biomedical Engineering School of Medicine Tehran University of Medical Sciences Tehran Iran; ^2^ Radiation Oncology Research Centre Cancer Institute Tehran University of Medical Sciences Tehran Iran; ^3^ Radiotherapy Physics Department Najaf Teaching Hospital Najaf Iraq; ^4^ Medical Radiation Research Center Central Tehran Branch Islamic Azad University Tehran Iran

**Keywords:** deformable image registration, dose distribution, kVCBCT, optical flow, radiotherapy

## Abstract

**Purpose:**

Kilovoltage cone beam computed tomography (kVCBCT)‐guided adaptive radiation therapy (ART) uses daily deformed CT (dCT), which is generated automatically through deformable registration methods. These registration methods may perform poorly in reproducing volumes of the target organ, rectum, and bladder during treatment. We analyzed the registration errors between the daily kVCBCTs and corresponding dCTs for these organs using the default optical flow algorithm and two registration procedures. We validated the effectiveness of these registration methods in replicating the geometry for dose calculation on kVCBCT for ART.

**Methods:**

We evaluated three deformable image registration (DIR) methods to assess their registration accuracy and dose calculation effeciency in mapping target and critical organs. The DIR methods include (1) default intensity‐based deformable registration, (2) hybrid deformable registration, and (3) a two‐step deformable registration process. Each technique was applied to a computerized imaging reference system (CIRS) phantom (Model 062 M) and to five patients who received volumetric modulated arc therapy to the prostate. Registration accuracy was assessed using the 95% Hausdorff distance (HD_95_) and Dice similarity coefficient (DSC), and each method was compared with the intensity‐based registration method. The improvement in the dCT image quality of the CIRS phantom and five patients was assessed by comparing dCT with kVCBCT. Image quality quantitative metrics for the phantom included the signal‐to‐noise ratio (SNR), uniformity, and contrast‐to‐noise ratio (CNR), whereas those for the patients included the mean absolute error (MAE), mean error, peak signal‐to‐noise ratio (PSNR), and structural similarity index measure (SSIM). To determine dose metric differences, we used a dose‐volume histogram (DVH) and 3.0%/0.3 mm gamma analysis to compare planning computed tomography (pCT) and kVCBCT recalculations with restimulated CT images used as a reference.

**Results:**

The dCT images generated by the hybrid (dCT_H_) and two‐step (dCT_C_) registration methods resulted in significant improvements compared to kVCBCT in the phantom model. Specifically, the SNR improved by 107% and 107.2%, the uniformity improved by 90% and 75%, and the CNR improved by 212.2% and 225.6 for dCT_H_ and dCT_C_ methods, respectively. For the patient images, the MAEs improved by 98% and 94%, the PSNRs improved by 16.3% and 22.9%, and the SSIMs improved by 1% and 1% in the dCT_H_ and dCT_C_ methods, respectively. For the geometric evaluation, only the two‐step registration method improved registration accuracy. The dCT_H_ method yielded an average HD_95_ of 12 mm and average DSC of 0.73, whereas dCT_C_ yielded an average HD_95_ of 2.9 mm and average DSC of 0.902. The DVH showed that the dCT_C_‐based dose calculations differed by <2% from the expected results for treatment targets and volumes of organs at risk. Additionally, gamma indices for dCT_C_‐based treatment plans were >95% at all points, whereas they were <95% for kVCBCT‐based treatment plans.

**Conclusion:**

The two‐step registration method outperforms the intensity‐based and hybrid registration methods. While the hybrid and two‐step‐based methods improved the image quality of kVCBCT in a linear accelerator, only the two‐step method improved the registration accuracy of the corresponding structures among the pCT and kVCBCT datasets. A two‐step registration process is recommended for applying kVCBCT to ART, which achieves better registration accuracy for local and global image structures. This method appears to be beneficial for radiotherapy dose calculation in patients with pelvic cancer.

## 1 INTRODUCTION

Volumetric modulated arc therapy (VMAT) is important for managing patients with prostate cancer, providing a uniform dose distribution while maintaining a steep dose gradient. This allows high doses to be delivered to the target while minimizing exposure to critical organs.^1^ VMAT can potentially improve the therapeutic index compared with three‐dimensional conformal radiotherapy, provided that uniform radiation doses are consistently delivered to the treatment target.^2^, ^3^ In practice, achieving a uniform dose distribution to the treatment target while sparing organs at risk is challenging owing to factors such as interfractional anatomical fluctuations, Variations in rectum and bladder filling further complicate accurate treatment.^2^, ^4^, ^5^, ^6^ A feasible solution for overcoming these challenges is the use of day‐to‐day computed tomography (CT) images in radiotherapy to incorporate anatomical changes over time.^7^, ^8^, ^9^ Adaptive radiation therapy (ART) typically uses deformable image registration (DIR) to align planning CT (pCT) images to kilovoltage cone‐beam computed tomography (kVCBCT) images. This process involves calculating the voxel correspondence between pCT and kVCBCT scans to auto recontour regions of interest (ROIs), calculate the dose, and account for the patient's accumulated dose.^10^, ^11^, ^12^, ^13^

In practice, daily image‐guided radiotherapy uses linac unit (Elekta XVI onboard volumetric imaging or Varian's onboard imager) acquired kVCBCTs to verify the patient setup.^14^, ^15^, ^16^ These kVCBCT images can also be used for ART to compute daily doses, provided that the CT numbers are accurate.^17^, ^18^ Deformable image registration (DIR) and rigid image registration are used in ART for precise adaptation.^11^, ^19^, ^20^, ^21^, ^22^ Despite the lower image quality of kVCBCTs, DIR is used to achieve good dosimetric agreement between pCT and kVCBCT, as shown in studies using optical flow or b‐spline algorithms.^17^, ^18^, ^19^, ^23^

Various strategies have been used to quantify the accuracy of deformable pCT and kVCBCT image registration for dose calculations. Giacometti et al. compared the default DIR method with other methods, such as standard calibration curves for pCT and kVCBCT and density overriding.^17^ The default DIR method involves software‐based deformable registration of moving and target images, without correction. Moteabbed et al. validated a combination of DIR methods using a calibration curve for dose calculation.^19^ However, DIR is most effective with feature‐rich images with a clear correspondence between the moving and target images. Inspecting these visible features will help determine the “goodness of fit,” as they are primary targets for dose calculations and thus the most relevant locations. However, anatomical images often contain subregions with low contrast or atypical anatomy, complicating DIR accuracy. Atypical anatomy refers to structures that vary during treatment. The DIR algorithm cannot redistribute voxels in such regions because there is little local information. Therefore, significant deformations or unclear boundaries between structures may lead to less accurate DIR accuracy. The pelvic region, in particular, experiences greater interfractional organ motion and deformation than that observed in other sites, such as the head and neck area.^24^ Previous studies have not investigated these concerns by including multiple patients with atypical anatomy.^25^, ^26^, ^27^

Currently, kVCBCT‐guided ART uses daily deformed CT (dCT) images automatically created by deformable registration algorithms. These algorithms may perform poorly in reproducing the variable volumes of the target organ, bladder, and rectum during treatment. This study analyzed mapping errors and dosimetric effects of the target organ, bladder, and rectum between daily kVCBCTs and corresponding dCT images using two developed registration methods.

This study used an in‐house algorithm and DIR processes to perfom deformable registration betw pelvic pCT and kVCBCT images. Section 2 presents solutions to the identified issues. The accuracy of rectal, bladder, and target volume mapping between pCT and kVCBCT images was evaluated, and the dosimetric impact of these inaccuracies in the prostate ART workflow was estimated. This evaluation is essential for mapping inaccuracies in rectal, bladder, and target volumes and adjusting ART workflows.

## 2 METHODS

This work compares the performance of three DIR methods developed to calculate doses based on kVCBCT images.

### 2.1 Patient cohort and data

pCT and kVCBCT images of a computerized imaging reference system (CIRS) phantom (Model 062 M) and 11 patients with prostate cancer were acquired to compare the ability of default registration and the developed DIR‐based methods for handling atypical anatomy.

pCT images were acquired using a Philips CT scanner with acquisition parameters of 120 keV and 213 mA for 470 s. The pCT images of the phantom and patients were reconstructed with voxel sizes of 1 × 1 × 3 mm^3^ and 0.9 × 0.9 × 2 mm^3^ in the x, y, and z vector directions, respectively. The volumes of the pCT images for the phantom and patients were 406 × 406 × 37 and 512 × 512 × 168, respectively.

kVCBCT images were acquired before treatment using the Versa‐HD Elekta XVI onboard volumetric imaging system, with prime factor values of 120 keV and 64 mA over 40 s. The kVCBCT images of both the phantom and the patients were reconstructed with a voxel size of 1 × 1 × 3 mm^3^. The volumes of the kVCBCT images for the phantom and patients were 500 × 500 × 132 and 410 × 410 × 84, respectively. One pCT image and two kVCBCT images per patient were used because additional kVCBCT images revealed discrepancies in the volume of target and organ‐at‐risk contours. These volume differences were used to resimulate computed tomography (rCT) for the five patients in this study.

All the images were preprocessed using the open‐source, computational environment for radiotherapy research (CERR) software before rigid and deformable registration.^19^ Deformable registration software requires the target kVCBCT image to be smaller than a moving pCT image. In our study, the kVCBCT images were relatively smaller in some cases and larger in others. Therefore, the treatment table was manually segmented from all the images to remove unnecessary content. Compared with kVCBCT, pCT has a larger range of superior‐inferior patient volumes. To standardize the image size and reduce computational time, both sets of images were cropped and downsampled to (1, 1, 2) in the (x, y, z) direction.

The datasets for the five patients included three cases of prostate beds and lymph nodes and two cases of prostate beds, seminal vesicles, and lymph nodes. For these adaptive pelvic treatments, two‐, three‐, or four‐arches VMAT configurations were used to reduce the on‐couch calculation time. Patient‐specific prescription regimens and treatment techniques are summarized in Table 1. According to Lemus et al., the DIR incorrectly maps the air volumes during treatment.^28^ To overcome this problem, we used the detection and filling method proposed by Yang et al.^29^ This method comprises three steps.

1. Producing a binary mask image (M_1_): In this step, M_1_ is produced by detecting all voxels within the CT volume (I_original_) with Hounsfield unit (HU) values below 800, which are classified as gas pockets. These voxels are marked with a value of 1, while all other voxels are assigned a value of 0.
2. Converting M_1_ to a painting image (I_painting_): Once M_1_ was produced, the CT numbers of all gas pocket voxels were updated by setting them to 1060. I_painting_ was then smoothed using a Gaussian low‐pass filter to obtain a smoothed painted image (I_painted smoothed_).
3. Blurring I_painting_ and its boundaries to match the bowel with those without gas pockets: This final step was achieved by extending M_1_ by two voxels and filtering the extended image (M_2_) using a Gaussian low‐pass filter (M2_filtered_)_._ Finally, to obtain a smooth transformation of the gas pocket boundaries with the original CT image outside the pockets, interpolation was applied between I_original_ and I_painted smoothed_ while using M_2 blurred_ as the per‐voxel interpolation parameter.

**TABLE 1:** Summary of patients' clinical characteristics and treatment features.

Site	Patients	Target dose	Fraction number	Technique	Treatment
Prostate bed and lymph nodes	2	70	30−35	VMAT	4 and 4 arches in for each patient, respectively.
Prostate, seminal vesicles, and lymph nodes	3	70	30−35	VMAT	2, 4, and 3 arches in for each patient, respectively.

An experienced radiation oncologist contoured the treatment target, bladder, rectum, seminal vesicles, and both femoral heads on the pCT scans and every kVCBCT scan to study a single fraction for each patient. Contour and dose calculations were performed using the MONACO treatment planning system (Elekta, Crawley, UK).

The clinical target volume (CTV) was defined based on the patient's risk level. For intermediate risk, the CTV included the prostate and the first 10 mm of the proximal seminal vesicles closest to the prostate. For high‐risk patients, the CTV included the prostate and the first 20 mm of the proximal seminal vesicles closest to the prostate. For the planning target volume (PTV), all sides of the prostate were margined with a 5‐mm margin, except posteriorly, where a 3‐mm margin was used. Contours were drawn around the bladder, small bowel, rectum, and femoral head to identify at‐risk organs.

### 2.2 Image registration

This study evaluated registration methods to account for atypical anatomy. The deformed images were compared with intensity‐based registration. These methods use algorithms, namely, b‐spline and optical flow, which improve the consistency of the pixel intensity and subsequently achieve an acceptable level of accuracy in dose calculations.^19^, ^29^

#### 2.2.1 Hybrid deformable registration: b‐spline and optical flow

In patients with prostate cancer, the treatment target is the prostate, and the critical organs at risk are the femoral head, rectum, and bladder. Accurate registration of these organs is vital for ART. Registration errors are associated with differences in the subcutaneous fat, rectum, and bladder fill volumes between the pCT and kVCBCT images.

These errors can be explained as follows:

- Intensity‐based registration algorithms are affected by twisting of the tissues because intensities inconsistencies between CT and kVCBCT in abdominal cases result in substantial registration errors.^30^
- Registration algorithms incorrectly recognize the bowel contents adjacent to the bladder and rectum. Therefore, algorithms partially register bowel contents to the bladder.
- The low contrast of soft tissue in the pelvic region results in algorithms not differentiating the bladder from the prostate.^25^

Nonlinear registration uses splines to create smooth curves. The spline may be controlled by several control points (b‐splines), which can be used to estimate complex nonlinear transformations of images of the head, neck, chest, and pelvis. Most studies have succeeded in calculating transformations and propagating contours from pCT to kVCBCT using deformable vector fields (DVFs) with b‐spline algorithms.^31^, ^32^, ^33^ However, splines have not been investigated for propagating structures from pCT to kVCBCT. Owing to iterative optical flow (IOF) successfully propagating HU values, we combined intensity‐based and geometry‐based algorithms to develop a hybrid method. This in‐house algorithm integrates both b‐spline and IOF registration methods.

The primary software tools used were Deformable Image Registration and Adaptive Radiation Therapy (DIRART) (version 2, https://github.com/krishprince/dirart) and CERR.^29^, ^34^ We used an open‐source DIR tool in CERR to apply DIR between each patient's pCT and kVCBCT images. Both images were uploaded to the CERR code and independently aligned.

CERR can be configured in single‐ or multistage modes to apply various registration algorithms and control parameters. This tool optimizes specific parameters (such as cost function metrics and subsampling rates) for more accurate registrations. This study used a multiresolution method to implement the b‐spline deformation algorithm in three stages. CERR does not need to align scans manually because it has a translation stage that helps align images in the first stage if required. DIR registrations are then performed in two phases. In parallel with kVCBCT preprocessing, a similarity measure based on the sum of squared differences (SSD) was calculated instead of mutual information (MI), given the superior performance of SSD compared with MI.^19^

**FIGURE 1:**
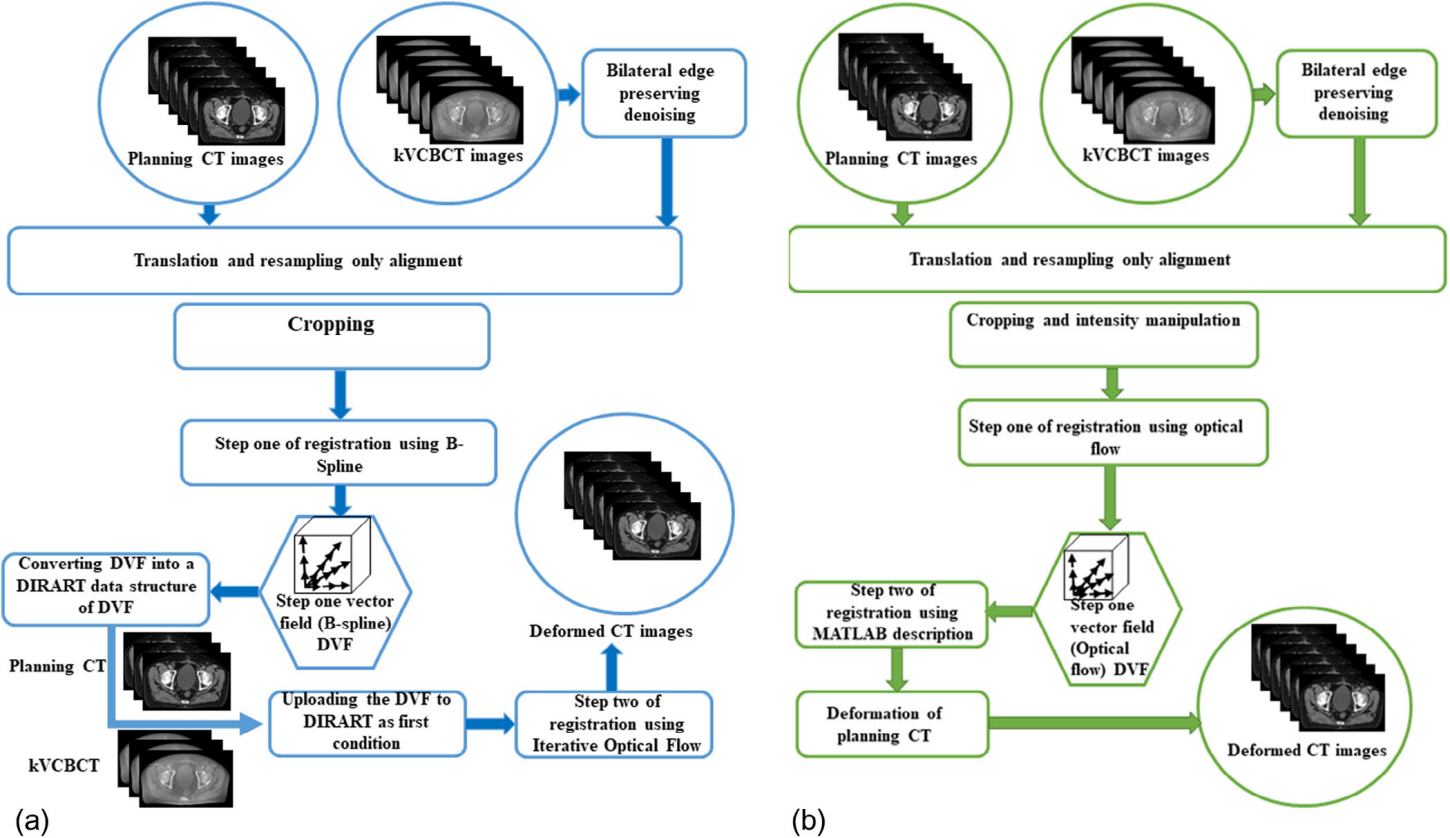
Workflow for deformable CT‐kVCBCT registration as determined by an in‐house hybrid algorithm and a two‐step process. Panel (a) shows a workflow for the hybrid registration method, and Panel (b) shows a workflow for the two‐step registration method.

The image registration workflow is shown in Figure 1 (Panel a). CERR registration was developed using spline methods (b‐spline and thin‐plate splines). The b‐spline registration method was applied to the CT scans to create a DVF inside the image space. Once the DVF was generated, it was converted into a DIRART data structure using MATLAB.

DIRART contains four DIR algorithms—optical flow, demand, level‐set, b‐spline, and newer inverse consistency algorithms—to provide consistent DVFs in both directions. The best‐deformed image resulting from an intensity‐based DIR was selected using DIRART, namely, an iterative optical flow. Therefore, the DVF matrix, which was previously calculated for the b‐spline algorithm in CERR, was used as the first condition for iterating the intensity‐based method to create the final deformed computed tomography (dCT_H_) images and the final DVF of the image of the patient's pelvis. The final DVF and dCT_H_ images had resolutions of 1 × 1 × 2 mm^3^ for the five patients.

#### 2.2.2 Two‐step DIR

We followed the procedure described by Yang et al.^25^ This aggressive method was applied because of challenged posed by the in‐house hybrid algorithm (explained in Subsection B.2.2.1) and registration errors associated with differences in rectum and bladder filling. This approach was achieved in three steps. First, the override approach was used before performing local registration. Experienced radiation oncologists contoured the treatment targets, critical organs, and subcutaneous fat in pCT and kV‐CBCT scans. The voxels of these structures were updated as follows:

(1) $$I-I_{\text{average}}+C\Rightarrow I$$

where I_average_ is the image's average intensity and is a constant set to 800, 1200, 800, and 1200 for the bladder, target, rectum, and subcutaneous fat, respectively.

Next, once the image intensity was overwritten, the first registration was performed using an optical flow algorithm. This involved a higher‐resolution stage with five iterations and ten passes, followed by a lower‐resolution stage with 30 iterations and 50 passes, and two multigrid stages with sigma = 1 mm for the Gaussian low‐pass filter on the DVF. Once the registration calculation was completed, the first DVF matrix was extracted through the MATLAB software. DIRART has a predefined MATLAB command that can deform an image based on the DVF from another registration. Consequently, the entire pCT image was deformed using the DVF from the previous step to produce a two‐step deformed image (dCT_C_). Figure 1 (Panel b) shows the workflow of the two‐step registration process.

### 2.3 Registration assessment

#### 2.3.1 Registration algorithm performance

The 95% Hausdorff distance (HD95) and the Dice similarity coefficient (DSC) were used according to TG‐132 to evaluate the accuracy of the DIR methods.^35^ The visual evaluation included checking the checkerboard images and the difference between the dCT and kVCBCT images for both the phantom and patients. The DSC quantitatively represents the volumetric overlap between annotations and segmentation outcomes. HD_95_ represents the maximum minimal distance of a contour on dCT to the nearest point on the other contour on kVCBCT. These metrics are described in Supporting File 1 (S1).

#### 2.3.2 Image quality

The phantom and patient datasets were used to assess whether the developed DIR methods enhanced the quality of dCT images. For the phantom, the evaluation indices included the identity function profile (IFP), the signal‐to‐noise ratio (SNR), the contrast‐to‐noise ratio (CNR), and uniformity. The mean and standard deviation (SD) were used to compute the bias of the CT number. The HU‐ED curves of the kVCBCT and dCT scans were evaluated in relation to those of the pCT. As a graphical representation of the CT number loss, we computed the IFP. The optimal DIR method correctly propagated all the HU values of pCT to kVCBCT, which caused the pixel intensity values of dCT to become similar to those of pCT. Therefore, an ideal registration method produces an identity function in the histogram. The closer the profile of the DIR method is to an identity function, the better the registration method. For patients, the evaluation indices included the calculation of the peak signal‐to‐noise ratio (PSNR), structural similarity index measure (SSIM), mean error (ME), and mean absolute error (MAE) for kVCBCT and dCT images versus pCT images. Supporting File 2 (S2) describes the image quality analysis methods.

### 2.4 Dosimetric evaluation

**FIGURE 2:**
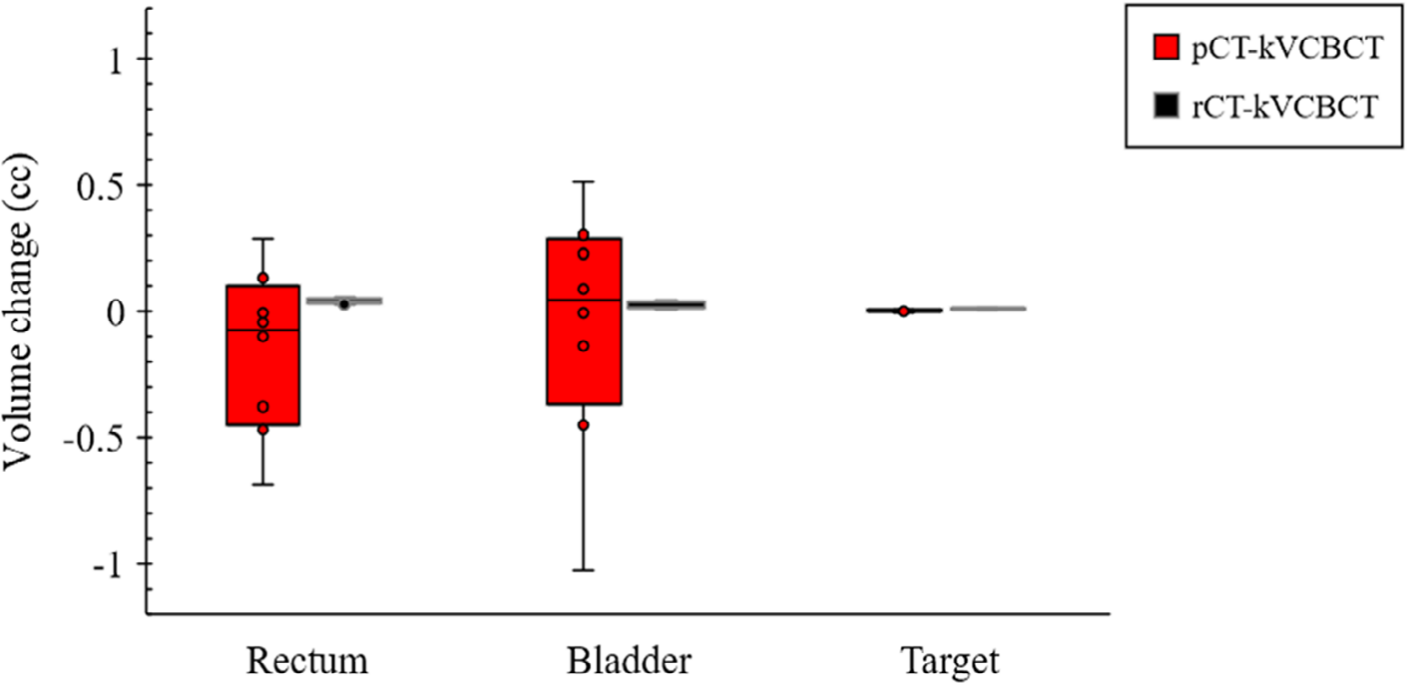
Box plots showing contour volumes differences between the target and organs at risk (rectum and bladder) from pCT to kVCBCT (red) and from rCT to kVCBCT (black) for the five patients included in this study.

New physician orders for replanning occur when there are setups or structural differences between pCT and kVCBCT, PTV under dosage, or overdosage to organs at risk based on dosimetric verification. Resimulated CT (rCT) was performed using the pCT system. A new plan was built from scratch, including ProSTEP immobilization, and it was designed as a kneepad and footpad with the same positioning as the previous one used in the pCT. Anatomical differences were considered, such as differences in bladder and rectal filling and the femoral heads laying in different positions.^23^ Consequently, based on a visual check, we chose patient datasets with anatomical features as close as possible to those seen on the kVCBCT images. The target and critical organs at risk were contoured on rCT, and their volumes were compared with those of kVCBCT. Therefore, the rCT images of patient datasets with more than a 95% difference in volume were chosen as reference images, as shown in Figure 2. As a result, only five of the eleven patients were included in this study.

Dose calculation outcomes were compared using dose‐volume histogram (DVH) analysis. These outcomes included calculating key plan characteristics of target and organ‐at‐risk structures specified in quantitative analyses of normal tissue effects in the clinic (QUANTEC)‐based dose metrics, including 98% PTV (D98%), the mean dose (Dmean) of the PTV, the maximum dose of the bladder, and the dose to 15% of the volume less than 75 Gy (V75 < 15%) of the rectum.^36^ The planning characteristics of the dCT_C_‐based plan were compared with those of DVHs computed using rCT plan data. Three CT modalities were used to validate the dose calculation results. These modalities included the kVCBCT‐based kVCBCT curve and two‐step‐based and rCT‐based CT curves. Dose calculations were performed using the Monte Carlo algorithm in the MONACO treatment planning system, which uses the same plan for each modality. In addition, the dose distributions obtained from kVCBCTs and dCTs were compared with those from rCT using the gamma function software (CERR). The results were considered passed when gamma indices were >95% and failed when gamma indices were <95%. Our gamma index calculations applied a 3% dose difference and 3 mm dose‐distance agreement.

## 3 RESULTS

### 3.1 Registration assessment

#### 3.1.1 Phantom study

**FIGURE 3:**
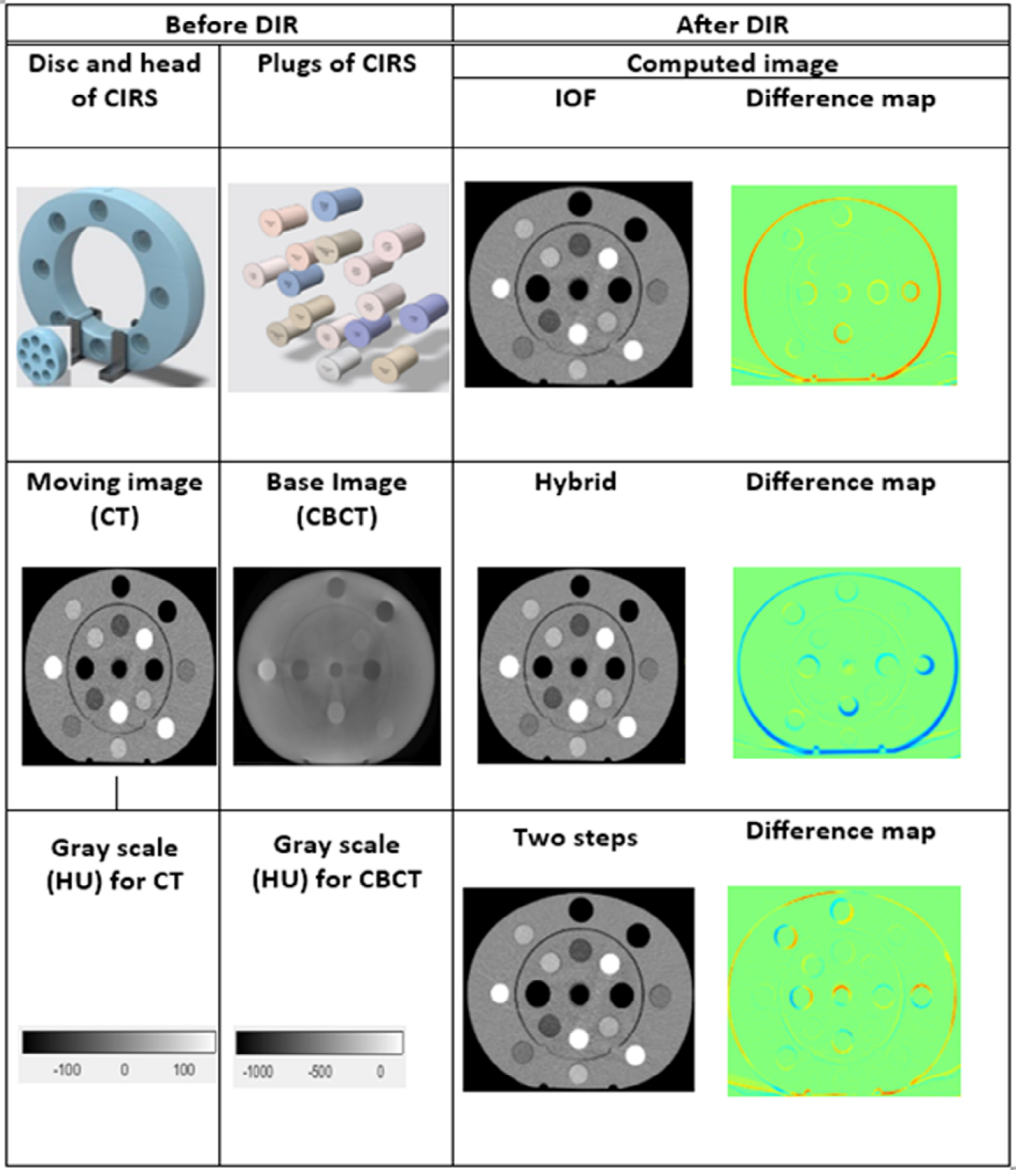
Assessment of DIR registration methods for phantom deformation. In this stage, the CT images are deformed to match the kVCBCT images. The first two columns present the moving images and base images before registration. The remaining columns demonstrate the deformed images guesstimated to the base image (kVCBCT). Unlike pCT, kVCBCT is associated with multiple sources of image degradation, such as stochastic (noise) and nonstochastic (scattering) deviations from the true image. Therefore, these image degradations cause the reconstructed kVCBCT images to differ from the object function.^37^ The reconstructed voxel value of kVCBCT subsequently suffers from inaccurately reconstructed voxel values. As a result, the CT numbers of kVCBCT are different from the CT numbers of pCT. Therefore, pCT and kVCBCT cannot be observed on the same scale for the HUs of the images. Abbreviation: HU, Hounsfield value.

**FIGURE 4:**
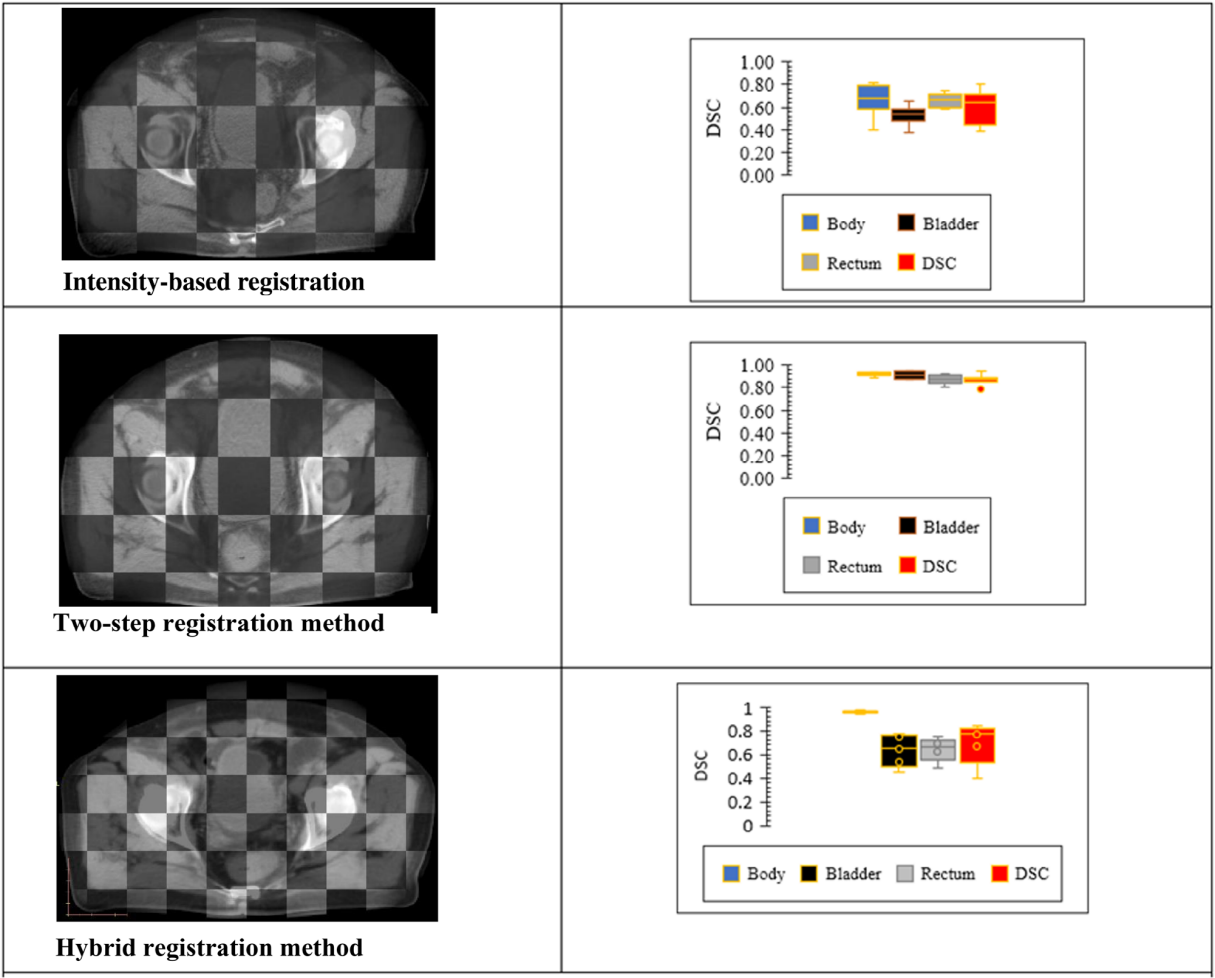
The average DSC for the intensity‐based, hybrid, and two‐step registration methods.

Figure 3 illustrates the visual assessment of the default and developed DIR processes to identify the method that correctly calculates intensities in addition to the disk, ring, and insert shapes. As shown in the figure, the IOF algorithm, in‐house hybrid algorithm, and two‐step registration process could compute the general shape of the disc, ring, and inserts of the CIRS phantom. Additionally, based on visual evaluation, the IOF, hybrid, and two‐step registration methods performed reasonably well in terms of HU values and shapes, matching the global (disc and head) and local (plug) shapes well (Figure 4).

#### 3.1.2 Patient study

Figure 4 shows registration samples for a prostate cancer patient. The figure illustrates that although the hybrid DIR method failed to register the overall image and critical organs, the two‐step DIR method (left column) successfully registered these components (left column).

In Figure 4, the average matching outcomes of critical organ volumes are summarized in the right column. For ART, the DSC should be no less than 0.8, according to Brock et al.^35^ The figure demonstrates that while the hybrid DIR method failed to register the treatment target and critical organs at risk, the two‐step DIR method showed significant improvement in volume matching. Compared with the hybrid method, the two‐step method improved the DSC by 22% (from 0.73 to 0.902). With all DSC values for the two‐step method exceeding 0.8 (Figure 4), the two‐step registration method achieves better registration than the hybrid method and is satisfactorily accurate for ART.

**FIGURE 5:**
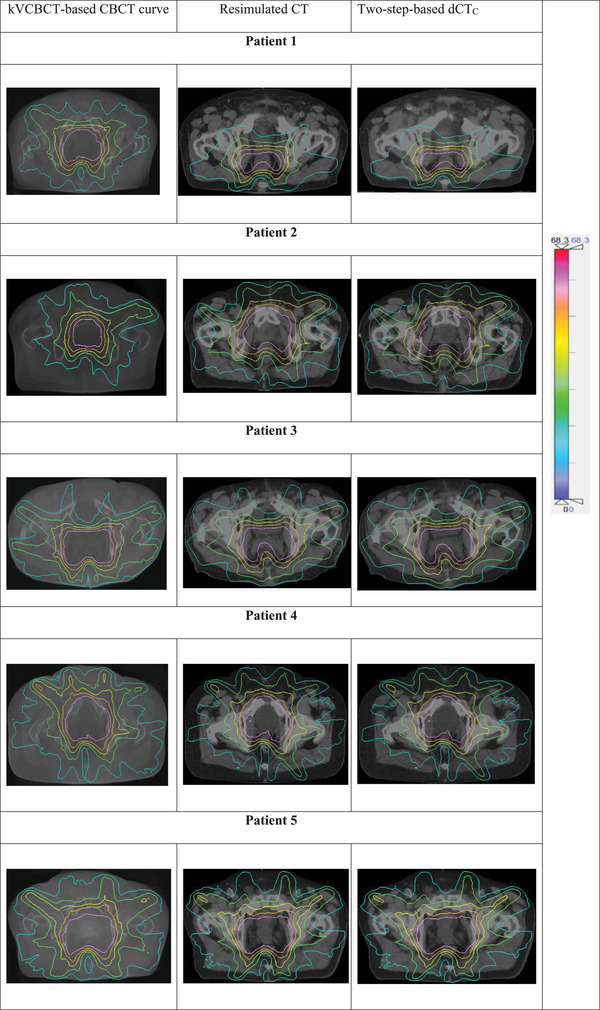
Dose distribution comparison of the kVCBCT, dCT, and rCT images of the five test patients.

Table 2 shows the HD95 (mm) results for contours in five prostate tumors. The results suggest that the two‐step registration performance is much better than the IOF and hybrid DIR methods. Compared with IOF and hybrid DIR, two‐step DIR reduces HD_95_ by 59% and 71% (from 7 and 12 to 2.9 mm), respectively.

**TABLE 2:** HD_95_ values (mm) for the contours of the five prostate tumors.

Registration method	Body	Bladder	Rectum	Target	Average value
IOF registration HD_95_ (mm)	10	7.2	6.5	4.3	7.0 ± 2
Hybrid registration HD_95_ (mm)	12	13	8.1	7.6	12 ± 2.8
Two‐step registration HD_95_ (mm)	2.8	2.7	2.9	2.9	2.9 ± 0.8

### 3.2 Image quality

#### 3.2.1 Phantom study

The image quality indices computed for the five CT modalities, namely, pCT, kVCBCT, IOF‐based dCT (dCT_IOF_), dCT_H_, and dCT_C_, are listed in Table 3. All the registration methods significantly improved the dCT images over the kVCBCT images. The SNR, uniformity, CNR, dCTH, and dCTC performances were better than those of kVCBCT for all the quality metrics. Compared with kVCBCT, dCT_C_ demonstrated an improved SNR (relative increases of 108%, 107.12%, and 107.25%), uniformity (relative decreases of 75%, 90%, and 95%), and CNR (relative increases of 220.2%, 212.3%, and 225.6%) for the IOF, hybrid, and two‐step DIR methods, respectively. Notably, the reconstructed kVCBCT images differ from the true object due to several factors degrading image quality, including nonstochastic (scattering) and stochastic (noise) deviations from the actual image.^37^ Therefore, this degradation leads to inaccurate reconstructed voxel kVCBCT values. As a result, the CT number values of the kVCBCTs fluctuated among different body tissues. For example, the mean value of adipose tissue was greater than that of muscle. This degradation led to negative SNRs, kVCBCT values, and CNRs. A comparison of dCT_H_ and dCT_C_ with kVCBCT IFP profiles is presented in Supplementary Figure S3 (A).

**TABLE 3:** Qualitative image quality results for the CIRS phantom.

Index	pCT	kVCBCT	IOF‐based dCT	Hybrid‐based dCT	Two‐step based dCT
SNR	3.50	−78.56	6.33	5.60	5.70
Uniformity	1.0%	2.0%	0.5%	0.2%	0.5%
CNR	7.48	−8.15	9.80	9.15	10.24

Abbreviations: CNR, contrast‐to‐noise ratio; dCT, deformed computed tomography; kVCBCT, kilovoltage cone beam computed tomography; pCT, planning computed tomography; SNR, signal‐to‐noise ratio.

#### 3.2.2 Patient study

The CT number profiles of the generated pCT, kVCBCT, and DCT images are compared and reported in Supplementary Figure S3 (B). The temporal changes in the volume of the ROI over the treatment course, accounting for some spatial trends in the CT number values between pCT and dCT, can be seen in the Supplementary Table S3. Table 4 summarizes the average quantitative image quality outcomes for the three CT modalities based on the patient data. Compared with kVCBCT, the IOF, hybrid, and two‐step DIR methods significantly improved all the evaluation indices. While the IOF algorithm improved the MAE by 97%, PSNR by 14.4%, and SSIM by 5.3%, the in‐house hybrid algorithm improved the MAE by 98%, PSNR by 16.3%, and SSIM by 1%, and the two‐step process improved the MAE by 94%, PSNR by 22.9%, and SSIM by 1% compared with kVCBCT. Compared with kVCBCT, the MAEs and MEs of the dCT_IOF,_ dCT_H_, and dCT_C_ images for the structures and patients are shown in the Supplementary Figure S3 (C and D).

**TABLE 4:** Quantitative results of image quality for prostate cancer patients.

Index	kVCBCT	Iterative Optical Flow	Hybrid	Two‐step
MAE	331.14 ± 66.420	7.9	6.16 ± 5.900	21.15 ± 8.900
PSNR (dB)	45.17 ± 1.330	51.71 ± 0.64	52.534 ± .000	55.52 ± 2.320
SSIM	0.94 ± 0.020	0.99 ± 0.004	0.99 ± 0.004	0.99 ± 0.004

Abbreviations: kVCBCT, kilovoltage cone beam computed tomography; MAE, mean absolute error; PSNR, peak signal‐to‐noise ratio; SSIM, structural similarity index.

## 4 ACCURACY OF THE DOSE CALCULATIONS

Compared to kVCBCT images, the dose accuracy significantly improved, with dCT_C_ images showing dose distributions nearly identical to those of rCT images. Figure 5 shows the side‐by‐side evaluation of the kVCBCT‐based, dCT_C_‐based, and rCT‐based dose calculations for the five test cases. Concurrently, the isodose lines of the kVCBCT image, especially the 20% (light blue isodose line), 30% (green isodose line), 60% (yellow isodose line), 90% (orange isodose line), 100% (rose isodose line), and 105% (red isodose line), significantly differed from those of the rCT image. The isodose line distribution of the dCT_C_ images is similar to that of the rCT images. Supporting File 4 (S4) shows an example of the DVH and local‐level dose metrics of kVCBCT, rCT, and dCT_C_ images for a dataset of patients who received radiation therapy to the prostate. As shown in Supplementary Figure S4 (A, B, and C), the DVH curves and local‐level dose differences for kVCBCT exhibited noticeable differences compared to rCT. Conversely, the DVH curves and local‐level dose differences of the dCT_C_ method were almost identical to those of the rCT method.

Table 5 shows the relative dose metric differences between the kVCBCT and two‐step methods compared with the rCT method, which is averaged over all patient datasets. Overall, the dose metric results of the kVCBCT method were considerably different from those of the rCT method; the difference is approximately 14.7%. The differences in the dose metrics between the kVCBCT and rCT methods were primarily reflected in the PTVs and organs at risk. The average D98% difference between the five PTVs for kVCBCT was 35.67%. However, the dose metrics from the two‐step method were closer to those of the rCT method. Compared with the rCT method, the two‐step method (0.4%) maintained more consistent outcomes.

**TABLE 5:** Comparison of kVCBCT and dCT_C_ relative doses to rCT averaged across all patients.

Critical organ	Dose metric	kVCBCT‐based CBCT curve mean ± SD (%)	dCT_C_ mean ± SD (%)
Treatment target	D_98%_	35.67 ± 28.20%	0.36 ± 0.20%
	D_mean_	4.65 ± 7.53%	0.09 ± 0.21%
	D_2%_	7.54 ± 3.92%	0.27 ± 0.18%
Bladder	D_max_	7.47 ± 6.54%	0.38 ± 0.23%
Rectum	V50 < 50%	21.20 ± 14.00%	0.09 ± 0.89%
	V60 < 35%	29.62 ± 15.41%	1.46 ± 1.27%
	V65 < 25%	13.01 ± 11.57%	0.53 ± 0.22%
	V70 < 20%	13.27 ± 10.15%	0.00 ± 0.29%
	V75 < 15%.	13.37 ± 9.08%	0.67 ± 0.37%
The average of all critical organ dose metrics		14.7%	0.4%

The gamma index values for the kVCBCT‐based treatment plan and dCT_C_‐based treatment plans are listed in Table 6. The distance‐to‐agreement (DTA) criterion and dose difference used in calculating the gamma function were set as 3 mm and 3%, respectively. As shown in Table 6, for the five patients, gamma function scores were lower than 95% for all kVCBCT‐based treatment plans, indicating poor agreement. Contrastingly, the pattern was reversed for dCT_C_‐based treatment plans, where the gamma functions were higher than 95% for all points. This highlights a discrepancy between kVCBCT‐based and rCT‐based treatment plans, while dCT_C_‐based treatment plans agreed with the rCT‐based treatment plans.

**TABLE 6:** Gamma index values for kVCBCT‐based and dCT_C_‐based treatment plans.

Patient number	kVCBCT‐based treatment plan	dCT_C_‐based treatment plan	*p*
1	0.01%	99.5%	<0.001
2	4.72%	99.8%	<0.001
3	0.5%	98.2%	<0.001
4	7.35%	100%	<0.001
5	6.23%	100%	<0.001

## 5 DISCUSSION

This study developed and evaluated two DIR approaches for scan propagation from pCT to kVCBCT images and compared them with an intensity‐based DIR method. These approaches include an in‐house hybrid algorithm and a two‐step DIR process.

This study developed two DIR methods, namely, an in‐house hybrid algorithm and a two‐step DIR process, to improve the registration of atypical anatomical structures and automatic segmentation. Our findings suggest that the in‐house hybrid and two‐step methods could reasonably propagate the CT number from pCT images to kVCBCT images with minimal error. Despite the reasonable success of the hybrid method in registering the disc plugs and ring shapes of the CIRS phantom, the hybrid method could not reproduce the geometry of the body and inserts, as shown in Figure 3 and Supplementary Figure S4 (A).

These two DIR methods were tested on a phantom model and patient datasets. The two‐step method achieved significantly better quantitative outcomes for all the segmentation metrics than the IOF and hybrid registration methods. The mean HD_95_ value was 7 mm for the IOF algorithm, 12 mm for the in‐house hybrid algorithm, and 2.9 mm for the two‐step DIR process in patients. Additionally, the mean DSC values were 0.6, 0.73, and 0.902 for the IOF algorithm, in‐house hybrid algorithm, and two‐step registration process among the patients, respectively, as shown in Table 2 and Figure 4. This suggests that while an optical flow combined with other override procedures can handle all DIR tasks, a point‐based algorithm combined with other algorithms cannot. Since this study investigated DIR accuracy, we did not examine the reasons for underperformance, as this was outside the scope of this study. This failure may be attributed to the mathematics inherent in the algorithm.

Compared with Chang et al., who used artificial intelligence and machine learning, our procedures, especially the two‐step DIR method, achieved good performance, with relative improvements to intensity‐based registration ranging from 22% to 71% for all segmentation metrics.^38^ Several studies have compared actual pelvic cancer patient treatment plans rather than a static phantom, which means that uncertainty due to patient physiological motion and anatomy variations have to be assessed. However, they did not evaluate registration accuracy, such as DSC and registration errors.^11^, ^19^, ^21^, ^22^, ^38^ In contrast, this study used the dCT_H_, dCT_C_, and dCT_IOF_ systems to evaluate registration accuracy.

Contouring information on the target and critical organs helped register these organs accurately using an image‐intensity based optical flow algorithm. Although several finite element models use structural information to register two images via boundary condition displacement and physical models of structural surfaces, using these nonlinear registration algorithms without combining them with an intensity‐based algorithm does not consider the image intensity information.^39^ Additional information for image matching of the contoured structures may be missed. Concerning geometric evaluation, the two‐step DIR methods succeeded in reproducing the geometry. It produced outcomes equal to the registration of the same modality, within a 3 mm registration error and 0.8−0.9 DSC in the pelvis. However, this method has advantages and disadvantages. For example, accurate voxel mapping can be generated on noncontoured objects and outside critical structures, and both structure contours and image intensity can be matched after intensity handling, which provides further image intensity information of the contours.

Compared with those of the kVCBCT images, all of the dCT image‐quality metrics revealed noticeable improvements in the phantom and patient studies for the hybrid and two‐step DIR methods, as shown in Supplementary File S3 and the Supplementary Figure S3 (A,B). Remarkably, all the metrics improved: SNRs (108%, 107%, and 107.2%), uniformity (75%, 90%, and 75%), and CNR (220.2%, 212.2%, and 225.6) for the IOF, hybrid, and two‐step registration methods in the phantom, respectively. For the patient data, MAEs (97%, 98%, and 94%), PSNRs (14.4%, 16.3%, and 22.9%), and SSIMs (5.3%, 1%, and 1%) improved for the IOF, hybrid, and two‐step DIR methods in the patient datasets, as shown in Tables 3 and 4, Supplementary Table S3, and Supplementary Figure S3 (C,D).

Finally, we created dCT (dCT_IOF,_ dCT_H_, and dCT_C_) images via the IOF, hybrid, and two‐step DIR methods. The IOF and in‐house hybrid algorithms failed to adequately reproduce the geometry adequately, whereas the two‐step registration process succeeded in reproducing the geometry of the patient dataset. The two‐step DIR process was further evaluated for dose accuracy via various strategies.

Quantitative DVH comparisons were conducted to evaluate the two‐step DIR process. In general, DIR produced dose distributions consistent with those of the rCTs (a dose‐to‐volume difference of less than 0.5% and no data points with gamma values less than 95%), as shown in Tables 5 and 6, Figure 5, and Supplementary Figure S4. Our results revealed that dCT_C_ images satisfied the requirements of clinical dose computations for prostate cancer patients. This method may contribute to a wide range of ART applications in the development of quality assurance techniques and clinical practice, such as predicting the radiation response of tumors via kVCBCT‐based radiomics and dose accumulation.^23^

In the two‐step registration process, the entire image volume and critical organs were registered, where the final DVF denoted the voxel mapping from the pCT image to the kVCBCT image. Therefore, the two‐step DIR method is feasible for different treatment adaptations. These include deforming the daily dose calculated on kVCBCT to pCT to accumulate the dose, triggering ART, and propagating contours from pCT to kVCBCT for structures not contoured on kVCBCT. However, this method has several disadvantages. The kVCBCT images have poor image quality, necessitating new organ and ROI contours. Without these methods, accurate registrations via a single‐intensity‐based registration algorithm are challenging, limiting the applicability of the proposed method.^25^

Furthermore, registration required 30 min to complete the process. Therefore, compared with radiotherapy, the time spent on radiotherapy is quite long. As a result, uncertainty factors such as organ volume variations and patient position changes may be obtained. These limitations are due to the use of locally applied, one‐off methods that use MATLAB scripts, which are labor‐intensive. With automatic contouring methods applied in commercial treatment planning systems, such as atlas‐based auto segmentation, it is possible to reduce manual contouring work and the process's duration.^29^ Thus, this method can be used for both online and offline ART.

Limitations explicitly associated with the two‐step registration process should be considered. First, we only studied prostate cancer cases to demonstrate the proposed procedure; therefore, other tumor sites should also be examined. In the future, the feasibility of dCT dose calculations should be investigated for online adaptive treatment planning. A further limitation of our study is that the kVCBCT images were of poor quality, causing inaccurate contouring of the structures.

## 6 CONCLUSION

This study compared two DIR methods with IOF algorithm for pCT images and daily kVCBCT images for curative ART. These are the hybrid and two‐step DIR methods. Although deformed pCT using the hybrid method proved unsuitable for ART, deformed pCT using the two‐step DIR method can be used for radiotherapy dose calculation. For patients with prostate cancer, better registration accuracy was achieved for the entire image, treatment target, and critical organs at risk using the two‐step DIR method. Consequently, kVCBCT can be used for dose calculations in patients with prostate cancer, with two‐step registration outperforming hybrid registration.

## AUTHOR CONTRIBUTIONS

The authors confirm their contributions to the study as follows: study conception and design: Hussam Hameed Jassim, Hassan Ali Nedaie, Ghazale Geraily, Ali Kazemian, Nooshin Banaee, and Danial Seifi Makrani; data collection: Hussam Hameed Jassim and Nooshin Banaee; analysis and interpretation of results: Hussam Hameed Jassim, Hassan Ali Nedaie, and Nooshin Banaee; draft manuscript preparation: Hussam Hameed Jassim and Nooshin Banaee. All the authors reviewed the results and approved the final version of the manuscript.

## CONFLICT OF INTEREST STATEMENT

The authors have no conflicts of interest to declare.

## ETHICS STATEMENT

This work was approved by the ethical committee of TUMS (reference code: IR.TUMS.IKHC.REC.1400.290).

## Supporting information

Supporting Information

Supporting Information

Supporting Information

Supporting Information

Supporting Information
