# Supplementary material for: Evaluation of the geometric and dosimetric accuracies of deformable image registration of targets and critical organs in prostate CBCT‐guided adaptive radiotherapy
Source: J Appl Clin Med Phys. 2024 Sep 13;25(11):e14490. doi: 10.1002/acm2.14490 (PMC11540054; doi:10.1002/acm2.14490)
Supplement: Supplementary file 1 — Supporting Information [file ACM2-25-e14490-s001.docx]

### **C.1.a. Phantom Study**

To use CT or kVCBCT for dose calculation, the scanner's Hounsfield unit (HU) must be related to the actual electron density. A CT phantom, the CIRS phantom, was used to calibrate pCT and kVCBCT.

**C.1.b. Patient Study**

This part of the study evaluated the capacity of DIR methods for managing deformed objects. For all cancer cases, volume masks of the target and critical organs were first contoured onto the dCT images by a clinical expert. Masks were copied into the kVCBCT image, and DSC and HD_95_ were calculated using CERR. DSC is defined by:

DSC = $\frac{2\left| X\cap Y \right|}{\left| X\left| +\left| Y \right| \right. \right.}$ (2)

where X represents the structure mask volume defined by the kVCBCT contour, Y represents the dCT structure mask volume defined by the dCT contour, and between X and Y is the joint operator ($\cap$).

This study used the CERR to compute the DSC and HD_95_ and evaluate the performance of the developed DIR [29]. The DSC score determines the segmentation reproducibility by measuring the overlap in the segmented images. The DSC ranged between 0 and 1. Higher the DSC, the greater is the overlap between the predicted and reference masks.^31^ Generally, a DSC value of 0.8-0.9 can be considered acceptable for adaptive radiotherapy.^30^ DSC has advantages and disadvantages; for instance, it can be straightforward to visualize but does not provide information about the direction of the error. Therefore, distance-based metrics were developed to resolve this issue, namely, HD_95_. Therefore, further data on the difference between evaluation and reference delineation can be obtained.^13, 33^ HD_95_ was calculated by:

d_H_ (X, Y) = max [d_XY_,d_XY_] = max {${max \atop x\in X}$ ${mind \atop y\in Y}(x, y)$, ${max \atop x\in X}$ ${mind \atop y\in Y}(x, y)$} (3)

where x represents set of the boundary points in X contour and y represents set of the boundary points in Y. 95th percentile HD (HD_95_), which is used to eliminate outlier subsets.
