# Supplementary material for: Evaluation of the geometric and dosimetric accuracies of deformable image registration of targets and critical organs in prostate CBCT‐guided adaptive radiotherapy
Source: J Appl Clin Med Phys. 2024 Sep 13;25(11):e14490. doi: 10.1002/acm2.14490 (PMC11540054; doi:10.1002/acm2.14490)
Supplement: Supplementary file 2 — Supporting Information [file ACM2-25-e14490-s002.docx]

**C.2.a. Phantom Study**

CNR was measured using the CIRS module (Figure 3). The CIRS module contains several targets made from different materials, such as bone 800, breast, liver, bone 200, lung exhale, and water. These components ranged from approximately −1000 HU to +1000 HU. For SNR and uniformity**,** circular regions of interest (ROIs) of 5 mm radius were used within the disc and ring components of the phantom. Five ROIs of the phantom water constitution were contoured to calculate the SNR, uniformity, and CNR. One was in the disc component and the others were in the ring component of the CIRS phantom. The mean and SD values of these regions were extracted using CERR software. The SNR of each ROI was computed as the ratio of its mean to SD. The final SNR values were the average of five SNR values. Higher values indicate better SNR. After computing the SNR of each ROI, the uniformity was computed as the percentage difference between any two mean values of the ROIs. The lower the value, the better is the uniformity. For CNR, ROIs of size 5 mm within the muscle and adipose tissue were used to measure the mean HU value.

The following formula was used to calculate the CNR:

CNR = $\frac{\mathrm{Mean}_{\mathrm{Muscle}}-\mathrm{Mean}_{\mathrm{Adipose}}}{\mathrm{Std}_{\mathrm{Muscle}}+\mathrm{Std}_{\mathrm{Adipose}}}$ $\times$ 2 (4)

where Mean_Muscle_ and Std_muscle_ are the mean and SD of the voxel values in the muscle, respectively, and Mean_Adipose_ and Std_Adipose_ represent the mean and SD of the voxel values in the adipose inserts. The greater the magnitude of the CNR values, greater the low-contrast visibility.

**C.2.b. Patient Study**

The test datasets were used to validate the enhancement of dCT over kVCBCT caused by the developed DIR methods. The pCT images were used as reference images. The improvement in dCT image quality was qualitatively and quantitatively evaluated. The former included a side-by-side evaluation of dCT against kVCBCT and pCT images, and a comparison of the CT number profiles. The latter included the calculation of the peak signal-to-noise ratio (PSNR), structural similarity index measure (SSIM), mean error (ME), and mean absolute error (MAE) for kVCBCT and dCT versus pCT images.

PSNR is used to measure the representation accuracy, where it represents the signal's maximum power against its noise power. Logarithmic decibel (dB) is used to express the PSNR. Higher the PSNR, better the image quality and lesser the distortion. This is expressed by:

PSNR = 10. log_10_$\left( \frac{\mathrm{MAX}_{I}^{2}}{\frac{1}{\mathrm{xy}}\sum_{i = 0}^{x - 1} \sum_{j = 0}^{y - 1} \left[ I \left( i, j \right) - K(i, j) \right]^{2}} \right)$ (5)

where MAX_I_ is the probable higher value of an image element (I) and x and y are the image dimensions in the vector direction (x, y).

SSIM is used to measure the similarity of an image to a reference image for two-dimensional objects. Therefore, it was calculated for each corresponding CT and kVCBCT image, and averaged. The SSIM values ranged between 0 and 1. Lower the SSIM value, lower the image distortion. The SSIM is defined by:

SSIM (x, y) = $\frac{(2m_{x}m_{y}+c_{1})(2s_{\mathrm{xy}}+c_{2})}{\left( 2m_{x}^{2}+m_{y}^{2}+c_{1} \right)(2s_{x}^{2}+s_{y}^{2}+c_{2})}$ (6)

where m_x_ and m_y_ represent the means of x and y, respectively, the variances of x and y are $s_{x}^{2}$ and $s_{y}^{2}$, respectively, and s_xy_ represents the covariance of x and y. C_1_ and C_2_ are constant values. C_1_ was calculated as C1 = (k_1_L)^2^ and C_2_ was calculated as C2 = (k_2_L)^2^, where values of k_1_ = 0.01, k_2_ = 0.03, and L = 2^B^ −1.

The MAE measures the absolute value of the average error of the predicted value compared with the true values of the CT number. The MAE was calculated separately for each slice. Lower the MAE, more accurate is the predicted value. It is defined by:

MAE =$\frac{\sum_{i=1}^{n} \left\| \mathrm{CT}_{\mathrm{pre}}(x_{i})-\mathrm{CT}_{\mathrm{ref}}(x_{i}) \right\|}{n}$ (7)

where n represents the total elements, (x_i_) is the number, CT_pre_ is kVCBCT or dCT and CT_ref_ is pCT images, respectively.
