# Supplementary material for: Evaluation of the geometric and dosimetric accuracies of deformable image registration of targets and critical organs in prostate CBCT‐guided adaptive radiotherapy
Source: J Appl Clin Med Phys. 2024 Sep 13;25(11):e14490. doi: 10.1002/acm2.14490 (PMC11540054; doi:10.1002/acm2.14490)
Supplement: Supplementary file 3 — Supporting Information [file ACM2-25-e14490-s004.docx]

Figure (S3: A) compares the IFP of dCT_IOF,_ dCT_H_ and dCT_C_ with that of kVCBCT. It can be seen in the figure that while dCT with both hybrid and two-step registration methods generates an IFP close to the identity function (the ideal value) for the treatment target and critical organs at risk, kVCBCT generates an IFP noticeably divergent from the ideal value, indicating that there are kVCBCT HU values of pixels that are significantly different from pCT. Therefore, it is evident that the application of DIR can significantly improve the consistency of the CT numbers. Because the pCT was deformed and the kVCBCT would not show distortions, the pCT HU values in those areas (plots c and d) would be expected to be a straight line. However, in plot c, the lung exhale was not well registered when registering a rigid phantom. Therefore, parts of the surrounding voxels were included in the average values measured for lung exhalation. Therefore, the lung exhalation point deviated from the straight line.

| (a) | (b) |
| --- | --- |
| (c) | (d) |

Figure (S3: A): (a) Correlation of the CT number between pCT and intensity DIR. (b) Correlation of the CT number between pCT and kVCBCT scans. (c) Correlation of the CT number between pCT and dCT_H_. (d) Correlation of CT number between pCT and dCT_C_. These selected points represent the average values measured for lung inhalation, lung exhalation, adipose, breast, water, muscle, liver, and bone 200 and 800 from the phantom.

Figure (S3: B) shows the registration samples for a patient with prostate cancer. As shown in the figure, although the hybrid registration method failed to register the overall image and critical organs, they were generally well registered with the two-step registration method (left column).

In (figure S3:B, right column), the average matching outcomes of critical organ volumes are summarized. For ART, the DSC should be no less than 0.8, according to Brock et al. (2017) [30]. As shown in the figure, while the hybrid registration method failed to register the treatment target and critical organs at risk, volume matching using the two-step registration method was considerably improved. The two-step method improved the DSC by 22% (from 0.73 to 0.902) compared with the hybrid registration method. Because all the DSC values of the two-step registration method in Figure S3: B) were higher than 0.8, the two-step registration method achieved better registration than the hybrid registration method and was satisfactorily accurate for ART.

Three sets of HU profiles are plotted in (figure (S3: B)) to estimate the improvement in the HU values. The data were acquired from the corresponding lines shown in the left corner of the image (Figure S3: B). Line profile passes through regions of interest, namely fat, soft tissue, water, and bone areas, ranging between −1000 HU and 700 HU. Again, the hybrid and two-step registration method results showed that the kVCBCT HU values were improved and became similar to the pCT HU values in prostate cancer cases. [Temporal change](https://www.classicthesaurus.com/temporal_change/synonyms)s in the volume of the ROI over the treatment course, as shown in Table S3, or six-degree setup errors, including longitudinal, vertical, lateral, yaw, pitch, and roll errors in prostate cancer treated with VMAT, seem to be the reason for some spatial trends in CT numbers between pCT and dCT.

| 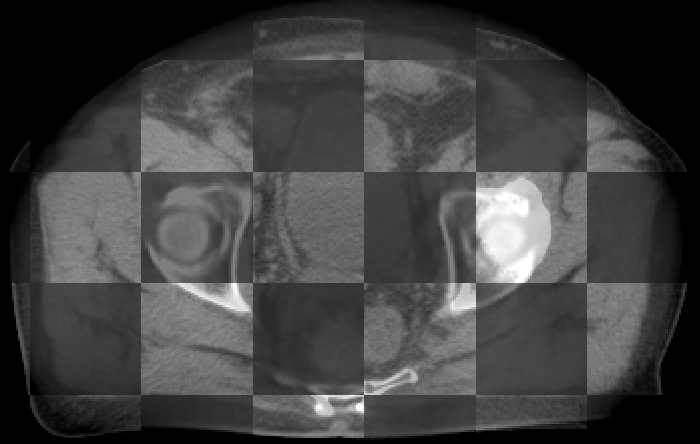  A |  |
| --- | --- |
| 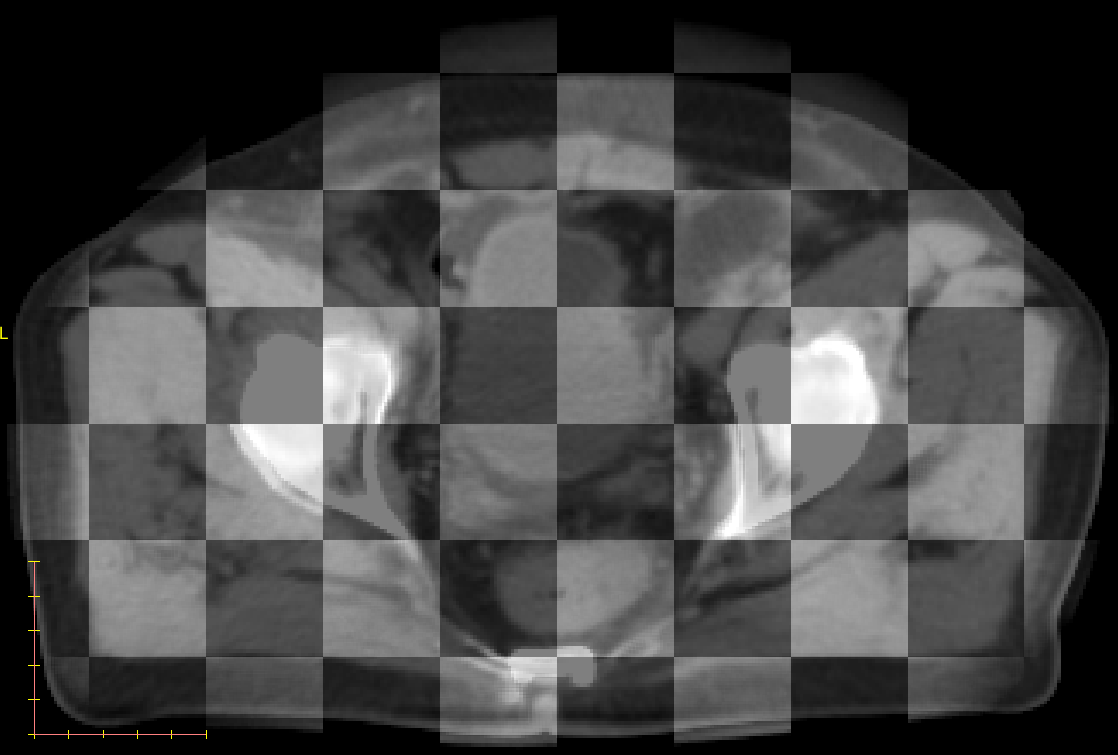  B |  |
| 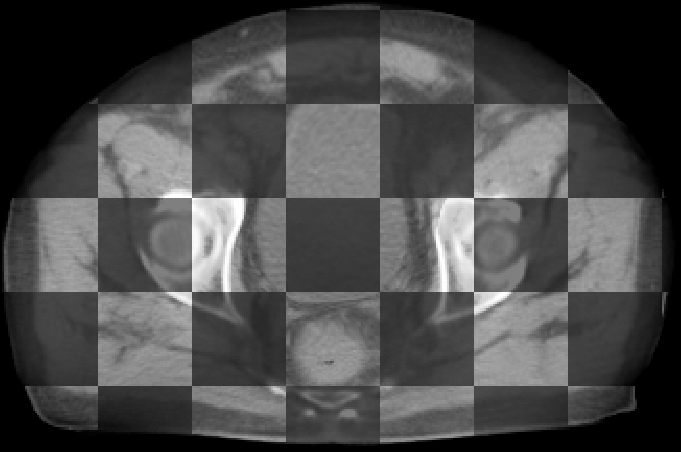  C |  |

Figure S3: B: Comparison of the CT number profiles of generated pCT, kVCBCT, and DCT images. The line profile passes through the air, soft tissue, and bone areas, ranging from −1000 HU to 2000 HU. Panel (A) shows the CT number profiles of generated dCT images of IOF against pCT and kVCBCT. Panel (B) shows the CT number profiles of generated dCT images of inhouse-hybrid against pCT and kVCBCT.

CT: computed tomography, IOF: Iterative Optical Flow, CBCT: Cone Beam Computed Tomography, dCT: deformed computed tomography.

Table (S3) shows the temporal changes in the critical target volumes for different imaging modalities.

|  | Planning target volume | | | | Rectum | | | | Bladder | | | |
| --- | --- | --- | --- | --- | --- | --- | --- | --- | --- | --- | --- | --- |
|  | pCT | dCT_IOF_ | dCT_H_ | dCT_C_ | pCT | dCT_IOF_ | dCT_H_ | dCT_C_ | pCT | dCT_IOF_ | dCT_H_ | dCT_C_ |
|  |  |  |  |  |  |  |  |  |  |  |  |  |
|  |  |  |  |  |  |  |  |  |  |  |  |  |
| Patient (1) | 273.2 | 273 | 109.1 | 273.6 | 63.40 | 63.4 | 62.7 | 57.70 | 65.13 | 65.13 | 62.5 | 139.4 |
|  |  |  |  |  |  |  |  |  |  |  |  |  |
| Patient (2) | 225.4 | 225 | 142 | 225.2 | 70.3 | 70.2 | 64.3 | 79.9 | 82.27 | 82.27 | 56.08 | 430.0 |
|  |  |  |  |  |  |  |  |  |  |  |  |  |
| Patient (3) | 414.8 | 415 | 87.2 | 415 | 430.0 | 430.03 | 61.8 | 209.112 | 48.15 | 48.15 | 412.8 | 63.40 |
| Patient (4) | 47 | 46.5 | 84.7 | 47.2 | 139.4 | 139.41 | 76.5 | 202 | 61.09 | 61.09 | 147.7 | 253 |
| Patient (5) | 242 | 241 | 172.1 | 240.1 | 253 | 252.98 | 42.9 | 176.1 | 238 | 237.96 | 243.2 | 228.2 |

In figure (S3: C), the MAE and ME values of kVCBCT are compared with those of the dCT_IOF,_ dCT_H_ and dCT_C_ images for the structures in all patient cases. Both MAE and ME values of kVCBCT and dCT fluctuated slightly. For instance, the MAE of kVCBCT ranged from approximately 203.87 380.35. Relative to MAE and ME of kVCBCT, dCT images of all registration methods of pelvic structures possess smaller MAE (2 to 13.6) and ME (-24.6 to 9.6), and MAE (15.37 to 28.38 HU) and ME (-0.49 to 11.14 HU) ranges for dCT_H_, dCT_H_ and dCT_C_ methods, respectively.

|  |
| --- |

Figure S3: C: Comparison of the MAE and ME between kVCBCT and dCT images by different registration methods for the test datasets. (a) compares MAE of kVCBCT to dCT_H_ and dCT_C_, (b) compares ME of kVCBCT to that of dCT_H_ and dCT_C_.

Figure (S3:D) compares the MAE and ME of kVCBCT relative to those of the dCT_IOF_, dCT_H_, and dCT_C_ images for all patient cases. As shown in the figure, in comparison with the kVCBCT images, the CT number values of the dCT images in all cases were considerably improved. While the MAE (maximum and minimum) ranges improved from (279.9828, 357.602) to (0.26, 10), (1.8, 13.6), and (15.4181, 35.4), the ME range improved from (-279.983, -357.602) to (-14.8, 7.8), (-5.5, 1.7), and (-9.2773, 2.024425) HU for dCT_IOF_, dCT_H_ and dCT_C_, respectively. The kVCBCT ME values were negative for all patients, and the dCT ME values were nearly consistent.

|  |
| --- |

Figure S3: D: Comparison of the MAE and ME between kVCBCT and dCT images with deformation methods for the test datasets. (a) compares MAE of kVCBCT to that of dCT, (b) compares ME of kVCBCT to that of dCT.

CT: computed tomography, kVCBCT: kilovoltage cone beam computed tomography.
