# Supplementary material for: Evaluation of the geometric and dosimetric accuracies of deformable image registration of targets and critical organs in prostate CBCT‐guided adaptive radiotherapy
Source: J Appl Clin Med Phys. 2024 Sep 13;25(11):e14490. doi: 10.1002/acm2.14490 (PMC11540054; doi:10.1002/acm2.14490)
Supplement: Supplementary file 4 — Supporting Information [file ACM2-25-e14490-s003.docx]

| (a)  (b)  (c) |
| --- |

Figure S4: DVH comparison of CBCT, dCT_C_, and resimulated CT images for target, bladder, and rectum (a). Local-level dose metrics of histogram comparison between resimulated CT and deformed CT (b). Local-level dose metrics of histogram comparison between resimulated CT and CBCT (c).

CT: computed tomography, PTV: planning target volume, CBCT: cone beam computed tomography.
