# Supplementary material for: Evaluation of the geometric and dosimetric accuracies of deformable image registration of targets and critical organs in prostate CBCT‐guided adaptive radiotherapy
Source: J Appl Clin Med Phys. 2024 Sep 13;25(11):e14490. doi: 10.1002/acm2.14490 (PMC11540054; doi:10.1002/acm2.14490)
Supplement: Supplementary file 5 — Supporting Information [file ACM2-25-e14490-s005.doc]

Table (S3) shows temporal changes in the volume of critical target volumes in different imaging modalities.

|  | Planning target volume | | | | Rectum | | | | Bladder | | | |
| --- | --- | --- | --- | --- | --- | --- | --- | --- | --- | --- | --- | --- |
|  | pCT | dCT_IOF_ | dCT_H_ | dCT_C_ | pCT | dCT_IOF_ | dCT_H_ | dCT_C_ | pCT | dCT_IOF_ | dCT_H_ | dCT_C_ |
|  |  |  |  |  |  |  |  |  |  |  |  |  |
|  |  |  |  |  |  |  |  |  |  |  |  |  |
| Patient (1) | 273.2 | 273 | 109.1 | 273.6 | 63.40 | 63.4 | 62.7 | 57.70 | 65.13 | 65.13 | 62.5 | 139.4 |
|  |  |  |  |  |  |  |  |  |  |  |  |  |
| Patient (2) | 225.4 | 225 | 142 | 225.2 | 70.3 | 70.2 | 64.3 | 79.9 | 82.27 | 82.27 | 56.08 | 430.0 |
|  |  |  |  |  |  |  |  |  |  |  |  |  |
| Patient (3) | 414.8 | 415 | 87.2 | 415 | 430.0 | 430.03 | 61.8 | 209.112 | 48.15 | 48.15 | 412.8 | 63.40 |
| Patient (4) | 47 | 46.5 | 84.7 | 47.2 | 139.4 | 139.41 | 76.5 | 202 | 61.09 | 61.09 | 147.7 | 253 |
| Patient (5) | 242 | 241 | 172.1 | 240.1 | 253 | 252.98 | 42.9 | 176.1 | 238 | 237.96 | 243.2 | 228.2 |
